# Harnessing two UDP‐glycosyltransferases from *Saponaria officinalis* toward heterologous production of endosomal escape enhancer saponins in *Gypsophila elegans* hairy roots

**DOI:** 10.1111/tpj.71109

**Published:** 2026-09-22

**Authors:** Tongtong Qu, Helga Schinkel, Tessa Moses, Kaylan Reddy, René de Vaumas, Stefan Schillberg, Alain Goossens, Elia Lacchini

**Affiliations:** ^1^ Department of Plant Biotechnology and Bioinformatics Ghent University 9052 Ghent Belgium; ^2^ VIB Center for Plant Systems Biology 9052 Ghent Belgium; ^3^ Fraunhofer Institute for Molecular Biology and Applied Ecology IME Forckenbeckstrasse 6 52074 Aachen Germany; ^4^ EdinOmics, RRID:SCR_021838 University of Edinburgh Max Born Crescent Edinburgh UK; ^5^ Extrasynthese Impasse Jacquard 69730 Genay France; ^6^ Institute for Molecular Biotechnology RWTH Aachen University Worringerweg 1 52074 Aachen Germany; ^7^ Department of Botany and Zoology Stellenbosch University Stellenbosch South Africa; ^8^ Department of Plant Molecular Biology University of Lausanne 1015 Lausanne Switzerland

**Keywords:** cell‐free biosynthesis, glycosylation, metabolic engineering, plant specialized metabolism, plant synthetic biology, recombinant protein, saponarioside, triterpenoid saponins

## Abstract

Soapwort (*Saponaria officinalis*) synthesizes a vast repertoire of specialized metabolites with bioactive properties. Among those, a specific type of highly glycosylated oleanane‐derived triterpenoid saponins has potential applications in the pharmaceutical industry due to their anticancer activity and enhancement of endosomal escape for targeted therapeutics. Saponarioside A and saponarioside B are representative of this group and the major bioactive saponins in soapwort. Recently, the enzymes involved in the biosynthetic pathway of saponarioside B have been characterized. SO1861, another soapwort saponin known for its endosomal escape properties, shares common structural features with these saponins, suggesting saponariosides may represent SO1861 precursors. However, the late‐pathway biosynthetic steps converting saponariosides into SO1861 remain unknown. Here, we report the identification and function of the uridine diphosphate (UDP)‐glycosyltransferase (UGT), UGT73EU1, and uncover an additional function of the previously characterized UGT73M2, both involved in the late biosynthetic pathway leading to SO1861 and its structural derivatives. We demonstrate their activity and utility through *in vitro* assays with recombinant UGT proteins produced in a cell‐free tobacco system. Furthermore, overexpression of *UGT73EU1* in hairy root cultures of *Gypsophila elegans*, a close relative of *Saponaria*, led to the accumulation of triterpenoid saponins typically not produced by this species. Our work showcases the potential of producing high‐value saponins in plants through genetic engineering approaches.

## Significance Statement

We characterize a previously unknown uridine diphosphate (UDP)‐glycosyltransferase (UGT), UGT73EU1, catalyzing the final glycosylation leading to the endosomal escape enhancer triterpene saponin SO1861, and show that UGT73M2 contributes to the same step through a previously unrecognized glucosyltransferase activity. We demonstrate their activity *in vitro*, using recombinant proteins produced in a plant‐based cell‐free system, and show that heterologous expression of *UGT73EU1* in *Gypsophila elegans* hairy roots leads to the accumulation of triterpenoid saponin compounds typically not produced by this plant, highlighting the potential of this enzyme for metabolic engineering of high‐value saponins in alternative plant production platforms.

## INTRODUCTION

Triterpenoid saponins are a diverse group of natural products found in higher plants and some marine organisms (Cárdenas et al., 2019; Dong & Qi, 2025; Kundu et al., 2025; Thimmappa et al., 2014). As many are synthesized by plants as defense compounds, their biosynthesis can be induced by jasmonate (JA) treatment (Lacchini & Goossens, 2020; Nguyen et al., 2022). These compounds, characterized by vast structural diversity, display a broad range of biological activities, including anti‐inflammatory and anticancer properties (Cárdenas et al., 2019; Goddard et al., 2024; Lacchini et al., 2023; Moses et al., 2014). A major example is QS‐21, a complex triterpenoid saponin derived from the bark of *Quillaja saponaria*, used as a vaccine adjuvant thanks to its potent immunostimulatory properties (Kensil et al., 1991; Martin et al., 2024; Pifferi et al., 2021).

In modern therapeutics, where targeted protein‐ and DNA‐based treatments primarily enter cells via endocytosis and become enclosed in endosomal vesicles, efficient cytosolic drug delivery remains a critical challenge (Ebrahimi & Samanta, 2023; Teo et al., 2021). Notably, specific highly glycosylated triterpenoids from *Saponaria officinalis* have been found to destabilize endosomal membranes, enabling the release of drugs from endosomal vesicles into the cytosol thereby enhancing therapeutic efficacy (Gilabert‐Oriol et al., 2015; Lacchini et al., 2023; Thakur et al., 2013). Triterpenoid saponins known for these properties are referred to as endosomal escape enhancers (EEEs). Triterpenoid saponins are present in various plant species across different tissues, including bark, leaves, stems, roots, and flowers. They are particularly abundant in members of the *Caryophyllaceae* family such as soapwort (*Saponaria* spp.) and baby's breath (*Gypsophila* spp.), as well as in the *Fabaceae* family like soybeans (*Glycine max* L.) and barrel medic (*Medicago truncatula* L.) (Dinday & Ghosh, 2023; Frechet et al., 1991; Henry et al., 1991; Moses et al., 2014; Sayama et al., 2012). In soapwort, the triterpenoid saponin sapofectosid (SO1861) has been identified as a transfection reagent and a potent endosomal escape agent (Fuchs et al., 2017; Kolster et al., 2024; Weng et al., 2015).

The biosynthesis of triterpenoid saponins spans multiple cellular compartments and involves diverse classes of enzymes. In the cytosol, oxidosqualene cyclase enzymes cyclize the linear sterol, 2,3‐epoxysqualene, into diverse cyclic compounds such as the pentacyclic triterpene β‐amyrin, which constitutes the backbone (i.e., aglycone) of many triterpenoid saponins. Next, on the outer surface of the endoplasmic reticulum (ER), membrane‐bound cytochrome P450 monooxygenases (P450s) selectively oxidize specific positions of the carbon backbone. Along the secretory pathway, uridine diphosphate (UDP) glycosyltransferases (UGTs) and acyltransferases (ATs) then decorate the triterpenoid scaffold with diverse sugar (d‐glucose, d‐galactose, d‐glucuronic acid, etc.) and ac(et)yl moieties, respectively (Dinday & Ghosh, 2023; Hao et al., 2025) Particularly, glycosylation is an essential step for bioactivity of saponins as it imparts amphipathic properties to the otherwise largely apolar aglycone.

Glycosylation is a common modification reaction that plays a crucial role in diversifying the physiochemical properties of plant natural products. The enzymes primarily responsible for this process belong to the carbohydrate‐active enzyme (CAZy) glycosyltransferase family 1 (GT1) and catalyze the transfer of sugar moieties to saponins and other specialized metabolites. However, a few UGTs from the GT2 family, such as cellulose synthase‐like (CSL) proteins, were recently shown to be implicated in saponin biosynthetic pathways (Chung et al., 2020; Jo et al., 2025; Jozwiak et al., 2020). Plant UGTs recognize their sugar donors through a highly conserved motif located in the C‐terminal region, known as the plant secondary product glycosyltransferase (PSPG) motif, which is shared throughout UGT families (Louveau & Osbourn, 2019). While most characterized plant UGTs utilize UDP‐glucose (UDP‐Glc) as their sugar donor, some have been identified to employ alternative sugars, such as UDP‐glucuronic acid (UDP‐GlcA), UDP‐galactose (UDP‐Gal), and UDP‐rhamnose (UDP‐Rha) (Gharabli & Welner, 2024; Louveau & Osbourn, 2019). Although UGTs typically exhibit low sequence identity, their tertiary structures are highly similar, all adopting a GT‐B fold composed of two catalytic domains formed by the N‐ and C‐terminal regions (Liu et al., 2023).

Beside *S. officinalis*, other species such as *Gypsophila elegans*, which also belong to the *Caryophyllaceae* family, produce triterpenoid saponins, such as gypsophilosid, which exhibit similar EEE properties (Lacchini et al., 2025; Sama et al., 2018). Gypsophilosid and SO1861 are structurally similar molecules sharing the same quillaic acid (QA) scaffold, a branched trisaccharide chain at the C‐3, and a branched tetrasaccharide at the C‐28 position. The only difference between these two compounds lies in the terminal moieties of the C‐28‐linked saccharide branches (Figure S1). Recent discovery of pathway genes for saponarioside B (SpB) has enabled the biosynthesis of its positional isomer SO1699, which may serve as a direct precursor to the pharmaceutically important EEE SO1861, as it lacks only the terminal glucose moiety (Figure S2) (Jo et al., 2025). In plant tissues, SO1861 is not the most prevalent saponin in *Saponaria* spp. Instead, other structurally similar saponins, such as SpB, are ubiquitously distributed in *Saponaria* tissues and their structural features suggest that they may act as intermediate or late‐pathway precursors to SO1861 (Jia et al., 1998; Jo et al., 2025).

Based on previously reported *S. officinalis* transcriptome sequence data from three organs (leaves, stems, and roots) upon mock or methyl jasmonate (MeJA) treatment, we used co‐expression analysis and functional characterization (Lacchini et al., 2025) to identify a hitherto uncharacterized UGT, UGT73EU1, as a new late‐pathway UGT and to assign an additional catalytic role to UGT73M2, both capable of converting late‐pathway intermediates into the pharmaceutically important EEE SO1861. Using two complementary approaches, we achieved SO1861 production: (i) *in vitro*, using cell‐free biosynthesis and bacterial expression systems to produce recombinant UGT proteins, and (ii) *in vivo*, through heterologous expression of novel *Saponaria UGT73EU1* in *G. elegans* hairy roots, which yielded a compound with accurate mass compatible with SO1861, typically absent in *Gypsophila*. These findings demonstrate the possibility of leveraging cross‐species metabolic engineering to produce valuable natural compounds, such as EEEs, in alternative production systems.

## RESULTS

### Discovery of genes involved in the biosynthesis of SO1861 in *S. officinalis*

**Figure 1:**
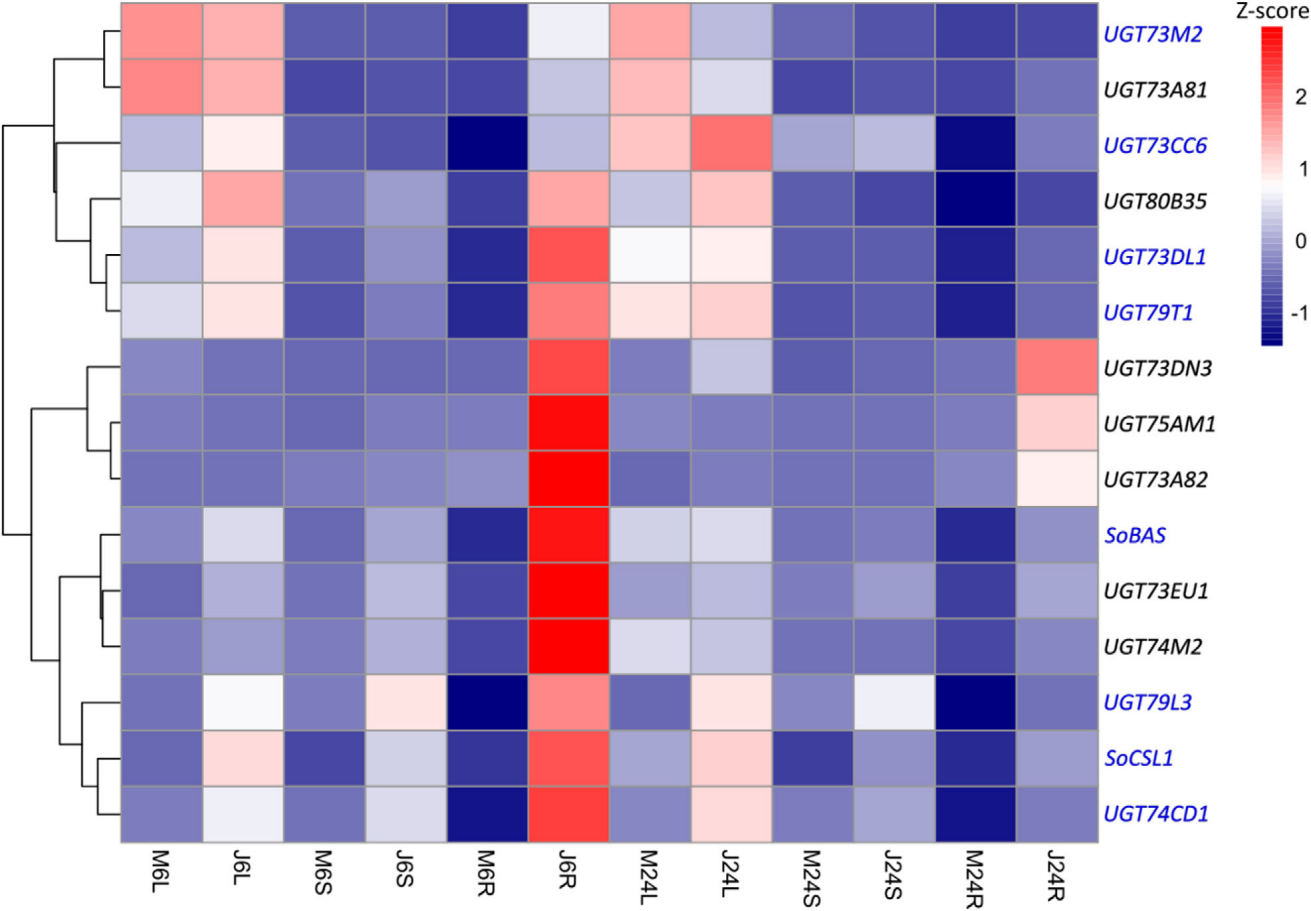
Heatmap displaying expression patterns of transcripts encoding candidate uridine diphosphate (UDP)‐glycosyltransferases (UGTs) and characterized triterpenoid saponin biosynthetic enzymes (in blue) in either mock‐ or methyl jasmonate (MeJA)‐treated *Saponaria officinalis* organs. *Saponaria officinalis* plants were treated with 50 μM MeJA (J) or ethanol (mock, M) and were sampled in triplicate at 6 and 24 h after treatment. Colors represent scaled expression values (*Z*‐score), where red indicates expression above the mean and blue indicates expression below the mean for each row. L = leaves, R = roots, and S = stems.

To identify UGTs potentially involved in the late glycosylation steps of SO1861, we reanalyzed transcriptome data previously published by our group (Lacchini et al., 2025). In that study, leaves, stems, and roots of hydroponically grown *S. officinalis* plants were treated with 50 μM MeJA or mock, and RNA was extracted at 6 and 24 h posttreatment in biological triplicates and sequenced. After normalization to transcripts per million (TPM), data clustering was conducted using the self‐organizing maps (SOM) algorithm to identify candidate SO1861 biosynthetic genes. Here we used the gene encoding β‐amyrin synthase (SoBAS) as a bait since it is the committed precursor enzyme in SO1861 biosynthesis. Several UGT genes were found in the same cluster as SoBAS and genes encoding cytochrome P450 (CYP450) known to be involved in the SO1861 biosynthetic pathway (Jo et al., 2025; Lacchini et al., 2025). Notably, most of the candidate UGTs varied across tissues, with the highest transcript abundance observed in MeJA‐treated roots at the 6‐h time point (Figure 1).

**Figure 2:**
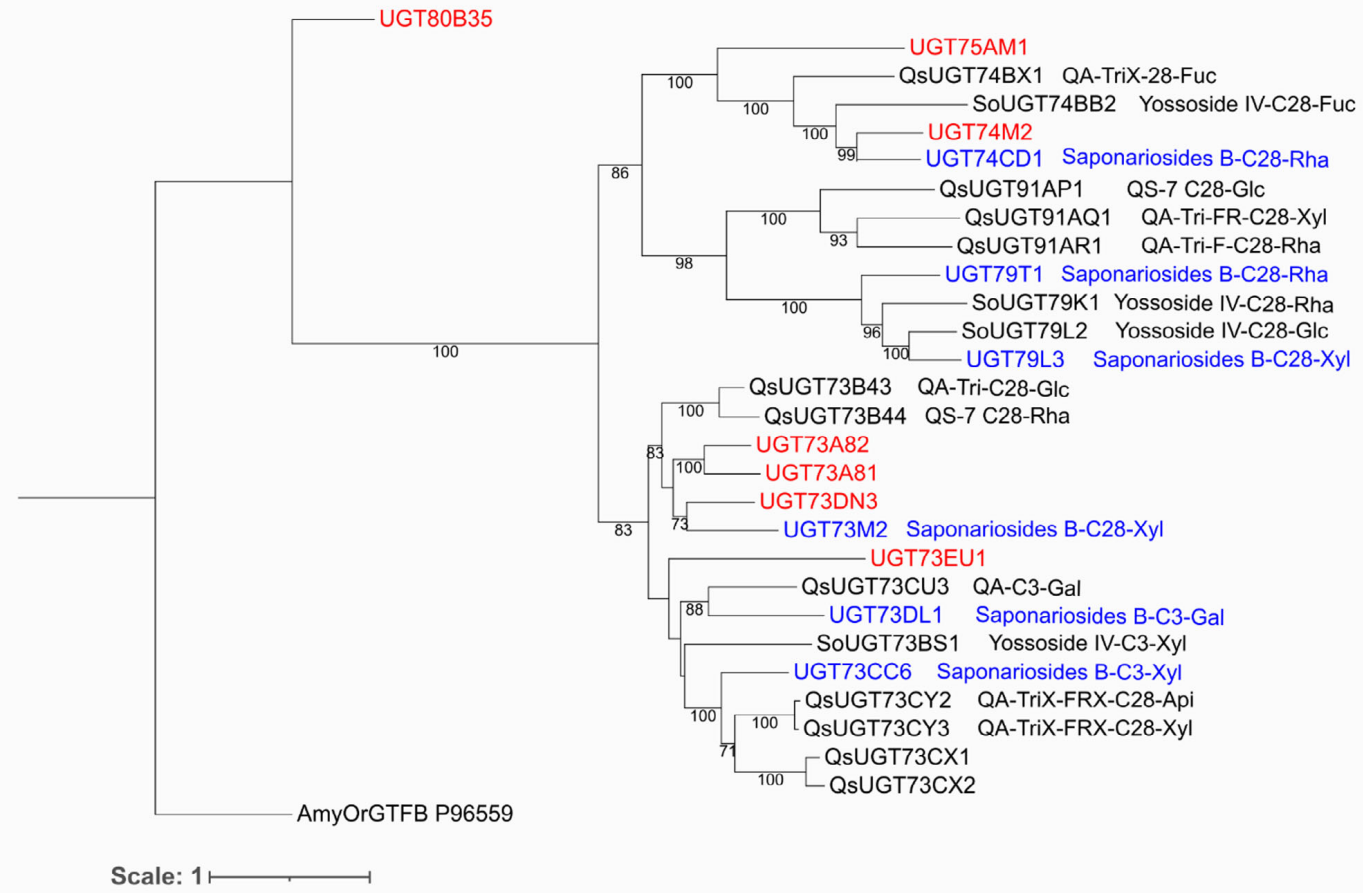
Phylogenetic analysis of translated protein sequences of *Saponaria officinalis* uridine diphosphate (UDP)‐glycosyltransferase (UGT) candidates. Uncharacterized *S. officinalis* UGT candidates are shown in red, while functionally characterized UGTs from the *S. officinalis* saponarioside biosynthetic pathway are highlighted in blue (Jo et al., 2025). Characterized UGTs involved in the biosynthesis of triterpenoid saponins, with endosomal escape enhancing properties, from *Quillaja saponaria* (Reed et al., 2023) and *Spinacia oleracea* (Jozwiak et al., 2020) are shown in black. The tree was constructed using IQ‐TREE (v2.4.0) pipeline on usegalaxy.eu, based on translated protein sequences aligned with MUSCLE, and the maximum likelihood (ML) tree was inferred with 1000 ultrafast bootstrap replicates (UFBoot) (the support value above 70% for branch points is shown). *Qs*, *Quillaja saponaria*; *So*, *Spinacia oleracea*.

Protein sequence comparison of *Saponaria* candidate UGTs with others already characterized as involved in triterpenoid saponin biosynthesis from other plant species showed that UGT80B35 forms a separate branch, indicating it may belong to a divergent lineage with unique substrate specificity. In contrast, UGT75AM1 and UGT74M2 cluster closely with characterized triterpenoid glycosyltransferases, implying possible involvement in triterpenoid or saponin glycosylation. Meanwhile, UGT73A82, UGT73A81, UGT73DN3, and UGT73EU1 group within the UGT73 subfamily, which is frequently associated with C‐28 glycosylation of triterpenoid saponins (Figure 2), suggesting that these UGTs may function similarly to QA glycosyltransferases. In addition to these uncharacterized UGTs, we also included UGT73M2, recently characterized in *Saponaria* as a xylosyltransferase responsible for transferring a xylose moiety to the C‐28 position of QA (Jo et al., 2025). Because plant UGTs are generally considered promiscuous in substrate recognition (Gharabli & Welner, 2024), UGT73M2 was included for further functional analysis as it may also accommodate structurally related compounds.

### Screening of candidate UGTs using an *in vitro* assay

**Figure 3:**
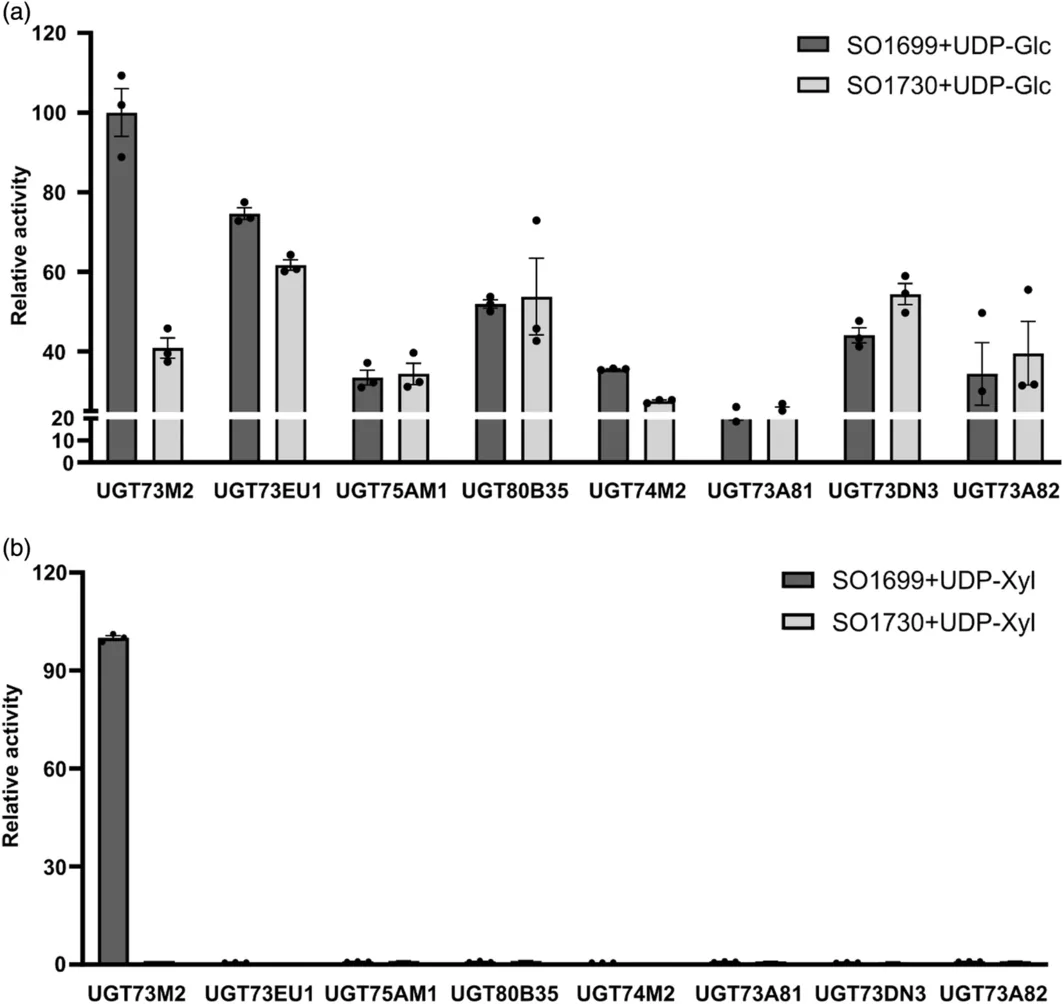
*In vitro* enzyme activities of recombinant candidate uridine diphosphate (UDP)‐glycosyltransferases (UGTs) assessed via the luminescence‐based UDP‐Glo™ assay. Candidate UGTs were tested with two saponin acceptor substrates (SO1699 and SO1730) and two sugar donors UDP‐Glc (a) and UDP‐Xyl (b). The relative enzyme activities were normalized to their corresponding controls lacking acceptor substrate. Error bars represent the mean ± SEM (*n* = 3 technical replicates).

To elucidate the final part of the SO1861 biosynthetic pathway, we decided to produce the eight candidate UGTs as recombinant proteins for functional characterization through *in vitro* enzymatic assays. The full‐length candidate genes were cloned from *S. officinalis* cDNA for expression; three were expressed as recombinant proteins in *Escherichia coli*, while the remaining five, which displayed poor expression in *E. coli*, were produced using a cell‐free expression system derived from tobacco BY‐2 cells (Figure S3) (Buntru et al., 2015, 2022). SpB is the major saponin found in *S. officinalis* (Jo et al., 2025). Here we speculate that its isomer, SO1699 (Figure S2), may be a direct precursor of SO1861, as they share the same triterpenoid structure and glycosylation pattern, with SO1861 having one additional terminal d‐glucose moiety. Interestingly, another saponin identified as SO1730 (C_78_H_122_O_42_) was detected by HPLC analysis from metabolite extracts of *S. officinalis* roots and differs from SO1861 by the absence of a d‐xylose group linked to the d‐quinovose group (Figure S2) (Moniuszko‐Szajwaj et al., 2016), and was therefore considered a possible alternative precursor for SO1861. Hence, SO1699 and SO1730 were purified from *S. officinalis* plant material and used as acceptor substrates for *in vitro* assays with the recombinant UGTs. Use of both acceptor substrates, in combination with candidate enzymes and UDP‐Glc or UDP‐Xylose (UDP‐Xyl) as sugar donors, allowed distinguishing which UGTs are involved in the production of SO1861 and/or SO1831 *in vitro* (Figure S2). The latter corresponds to a compound structurally analogous to SO1861 in which the terminal C28‐linked hexose (glucose) is replaced by the pentose (xylose). To this end, a preliminary screening of candidate UGTs was performed using UDP‐Glo glycosyltransferase (UDP‐Glo™) assays. This is a luciferase‐based assay that reports on the consumption of UDP‐sugars as donor substrates and the release of free UDP as a product. After the *in vitro* reaction, released free UDP is converted into ATP by a detection reagent, which is subsequently used by luciferase to generate bioluminescence. Luminescence can be quantified with a microplate luminometer enabling high‐throughput comparison of the enzymatic activities among candidate UGTs. As a result, we found that one candidate enzyme, UGT73M2, exhibited high activity with both UDP‐Xyl and UDP‐Glc and SO1699 as the acceptor substrate (Figure 3), pointing to a certain promiscuity toward different sugar donors. Conversely, UGT73EU1 displayed plasticity toward acceptors being capable of glucosylating (but not xylosylating) both SO1699 and SO1730 (Figure 3a) while demonstrating specificity for UDP‐Glc. Remaining candidate UGTs, like UGT74M2, UGT75AM1, and UGT73A81, showed a relatively lower catalytic activity toward both SO1699 and SO1730, whereas UGT73DN3 and UGT80B35 exhibited little preference for SO1730 (Figure 3a). With the exception of UGT73M2 on SO1699, the remaining UGTs did not show detectable activity toward UDP‐Xyl by UDP‐Glo™ assays (Figure 3b).

**Figure 4:**
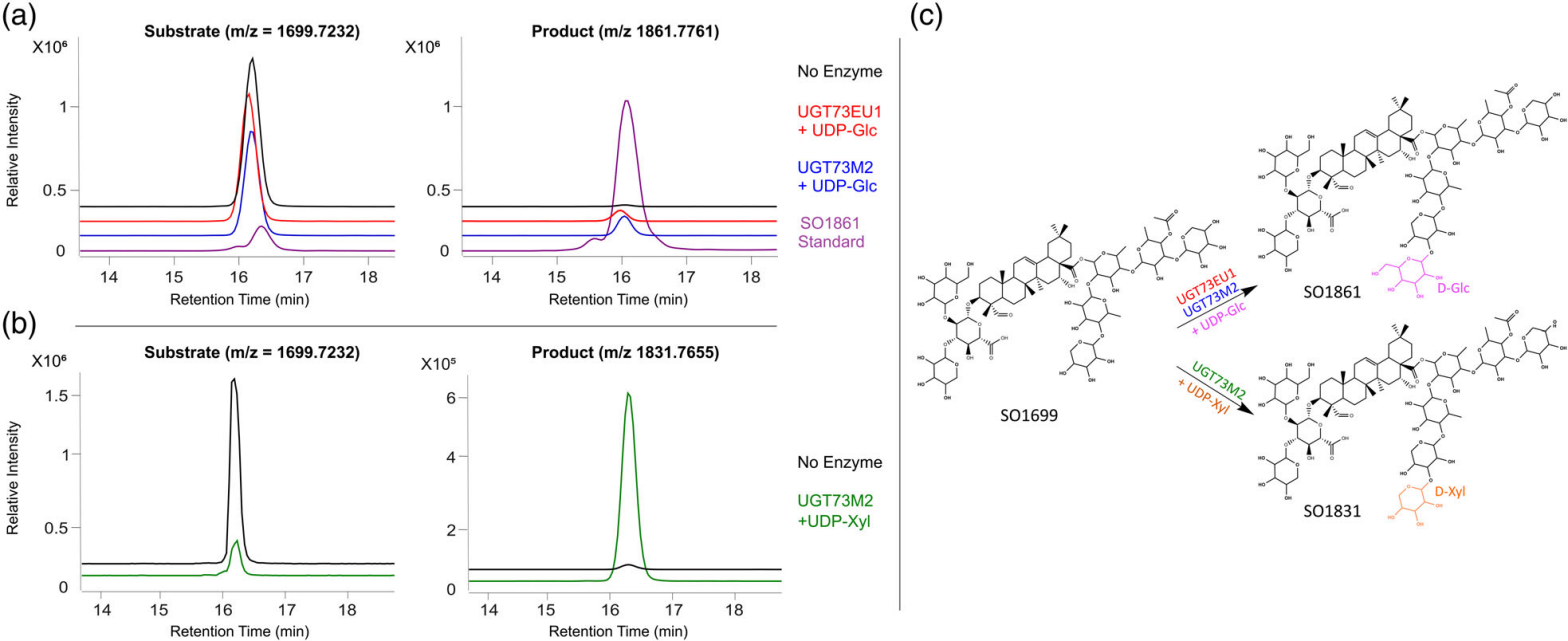
*In vitro* enzyme activities of recombinant candidate UGTs assessed by LC–MS. (a) Extracted ion chromatograms (EICs) of substrate SO1699 (*m/z* 1699.7232) and the product of SO1861 (*m/z* 1861.7761) peak in the presence of UDP‐Glc and UGT73EU1 or UGT73M2. The sapofectosid standard is plotted in purple. The *X*‐axis shows the retention time in minutes and the *Y*‐axis corresponds to the peak intensity of 1 × 10^6^. (b) EICs of substrate SO1699 (*m/z* 1699.7232) and the product SO1831 (*m/z* 1831.7655) peak in the presence of UDP‐Xyl and UGT73M2. The *X*‐axis shows time in minutes and the *Y*‐axis corresponds to peak intensity of 6 × 10^5^ for the product and 1.5 × 10^6^ for the substrate. MS and MS/MS fragmentation of SO1861 and SO1831 at 40 V, which are shown in the Figures S5 and S7, respectively. (c) Graphical overview of the conversion of SO1699 into SO1831 and SO1861 by *Saponaria* UGTs.

Next, to better resolve product identities and substrate specificities, we complemented the luminescence‐based assays by repeating the *in vitro* enzymatic reactions and analyzing the products by liquid chromatography‐mass spectrometry (LC–MS). These analyses confirmed that both UGT73EU1 and UGT73M2 converted SO1699 into SO1861, based on retention time, accurate mass, and MS/MS fragmentation patterns matching the SO1861 standards (Figure 4a; Figures S4 and S5). This finding is consistent with the UDP‐Glo™ assay results, highlighting that both enzymes can utilize UDP‐Glc as a sugar donor to form SO1861. When UDP‐Xyl was used instead, UGT73M2 produced a major compound with a retention time and mass spectrum consistent with SO1831 ([M − H] − *m/z* = 1831.7655), whose MS/MS fragmentation was consistent with the structure of SO1861 except for the substitution of the terminal hexose (glucose) by a pentose (xylose)on SO1699 (Figure 4b; Figures S6 and S7). This indicates that UGT73M2 can also catalyze xylosylation, thereby producing a compound (hereby referred to as *m/z* 1831) with features coherent with SO1831, a saponin naturally abundant in *S. officinalis*, presumably saponarioside A (SpA). Notably, our analyses suggest a broader substrate specificity by UGT73M2 than previously anticipated (Jo et al., 2025). In fact, UGT73M2 was found to act earlier in the pathway toward SpB, more precisely on the compound QA‐TriFRX (*m/z* 1379.6119) (Jo et al., 2025). Altogether, these results suggest that UGT73M2 has the potential to be active at different stages of the *S. officinalis* sapofectosid biosynthetic pathway. In addition, a minor product was detected when UGT73EU1 was incubated with SO1730 and UDP‐Glc, yielding a compound with mass [M − H] − = *m/z* 1891.7866. Although this *m/z* is compatible with the addition of a glucose moiety onto SO1730, further characterization of this product was not pursued due to its very low abundance.

None of the other UGT enzymes screened by the UDP‐Glo™ assay yielded detectable products by LC–MS when supplied with either UDP‐Xyl or UDP‐Glc as sugar donors (Table S1). This group included UGT80B35 and UGT73DN3, which nevertheless generated comparable signals to UGT73EU1 in the UDP‐Glo™ assays (Figure 3a), underscoring the importance of validating high‐throughput screening results by LC–MS. Importantly, the two UGTs that showed the strongest UDP‐Glo™ signals also demonstrated clear catalytic activity by LC–MS, highlighting the reliability and utility of luminescence‐based high‐throughput assays for preliminary screenings to identify promising candidates. Together, these results demonstrate that SO1861 and SO1831 are possibly formed through the complementary activities of UGT73EU1 and UGT73M2, with UGT73M2 displaying broader donor specificity and thus, by extension, a broader products profile.

### Subcellular localization of UGT73EU1 and UGT73M2 in *Nicotiana benthamiana* leaves

**Figure 5:**
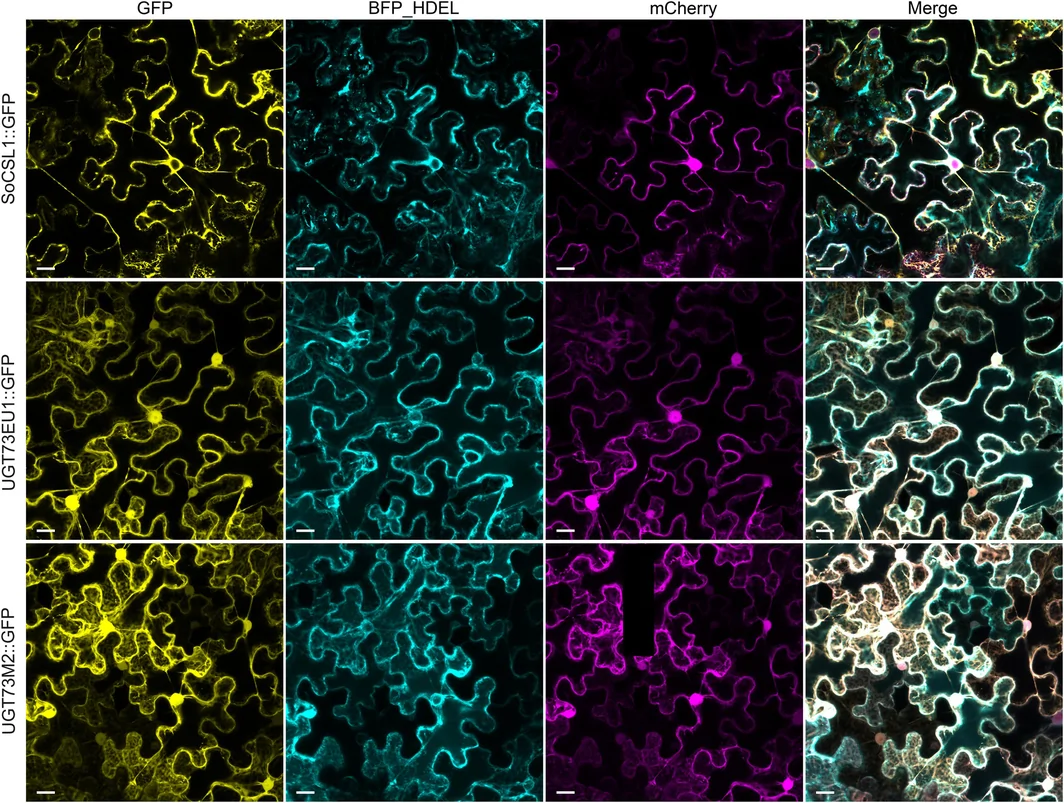
Confocal microscopy images show uridine diphosphate (UDP)‐glycosyltransferase (UGT)–green fluorescent protein (GFP) fusion proteins transiently expressed in *Nicotiana benthamiana* leaves, co‐expressed with BFP–HDEL, a resident endoplasmic reticulum (ER) lumen marker, and free mCherry as a cytosolic and nuclear marker. *Saponaria* UGT73EU1 and UGT73M2 localize to the nucleus and cytoplasm. SoCSL1, a glucuronic acid transferase from *Saponaria officinalis*, localizes to the ER and is shown for comparison (Jo et al., 2025). Panels display GFP (Yellow), BFP (Cyan), or RFP (Magenta) and merged channels. Scale bars, 20 μm.

Based on our enzymatic screening and LC–MS validation, UGT73EU1 and UGT73M2 emerged as the most promising candidates for EEE saponins. We therefore investigated the subcellular localization of UGT73EU1 and UGT73M2. Each enzyme was thus tagged with green fluorescent protein (GFP) at its C‐terminus. Confocal microscopy of *N. benthamiana* leaves expressing UGT73EU1‐GFP or UGT73M2‐GFP translational fusions together with free mCherry as a soluble protein control and ER‐localized Blue Fluorescent Protein (BFP‐HDEL) revealed that both UGTs colocalize with free mCherry in the cytosol and nucleus (Figure 5). This indicates that they are soluble nucleocytosolic proteins. As an additional control, we assessed the subcellular localization of cellulose synthase‐like 1 (SoCSL1) protein, which catalyzes the commitment of QA to EEE biosynthesis by the addition of the first sugar moiety (GlcA) to the 3‐O position (Jo et al., 2025). Consistent with the ER localization reported for related CSL proteins (Chung et al., 2020), SoCSL1:GFP localized to the ER network (Figure 5). Other previously characterized UGTs involved in the SO1861 biosynthetic pathway were localized to the cytosol and nucleus (Figure S8). Together, these results confirm that the first glycosylation step occurs on the ER membrane, along with the previous oxidations of the aglycone by CYP450s, while subsequent glycosylation steps of complex saponins are carried out in the cytoplasm.

### Biochemical characterization of UGT73EU1 and UGT73M2

To further characterize the biochemical features of UGT73EU1 and UGT73M2, we determined apparent kinetic parameters of the purified recombinant proteins toward their substrates and sugar donors using luminescence‐based glycosyltransferase assays (Figure S9). UGT73EU1 was tested with varying concentrations of the two putative acceptor substrates, SO1699 and SO1730, using UDP‐Glc as the sugar donor. The enzyme accepted both acceptors and followed Michaelis–Menten kinetics, with apparent Km values of 21.9 μM for SO1699 and 2.8 μM for SO1730 (Figure S9a,b). While UGT73EU1 displayed overall higher catalytic efficiency (kcat/Km) toward SO1730, its lower Km on SO1730 was also accompanied by a lower kcat, resulting in catalytic efficiencies of the same order of magnitude for both acceptors under the conditions tested. UGT73M2 was assayed with SO1699 as acceptor molecule in the presence of either UDP‐Glc or UDP‐Xyl, varying both donor and acceptor concentrations. Apparent Km values suggested tighter binding of UDP‐Glc (45.3 μM Km) than of UDP‐Xyl (155.7 μM Km) on UGT73M2 (Figure S9d,f). Interestingly, the apparent kcat of UGT73M2 toward SO1699 was approximately two orders of magnitude higher with UDP‐Xyl than with UDP‐Glc as donor (Figure S9c,e), indicating that although UDP‐Glc binds more tightly, the xylosylation reaction proceeds with faster turnover. This is consistent with the LC–MS analysis of the *in vitro* reactions, in which UGT73M2 product accumulation was predominantly observed using UDP‐Xyl rather than UDP‐Glc (Figure 4; Figures S4 and S6). Together, these findings highlight different kinetic strategies between UGT73EU1 and UGT73M2. The former accepts only UDP‐Glc and acts on both acceptors with slight preference for SO1730, while the latter configures as a donor‐promiscuous enzyme whose *in vitro* product profile is biased toward xylosylation. Such combination of donor and acceptor specificity coupled to differential turnover illustrates the versatility of triterpenoid UGTs and aligns with reported promiscuity of UGTs involved in plant specialized metabolism (Rahimi et al., 2019).

### UGT73EU1 and UGT73M2 catalyze the last glycosylation leading to SO1861 production *in vitro*

**Figure 6:**
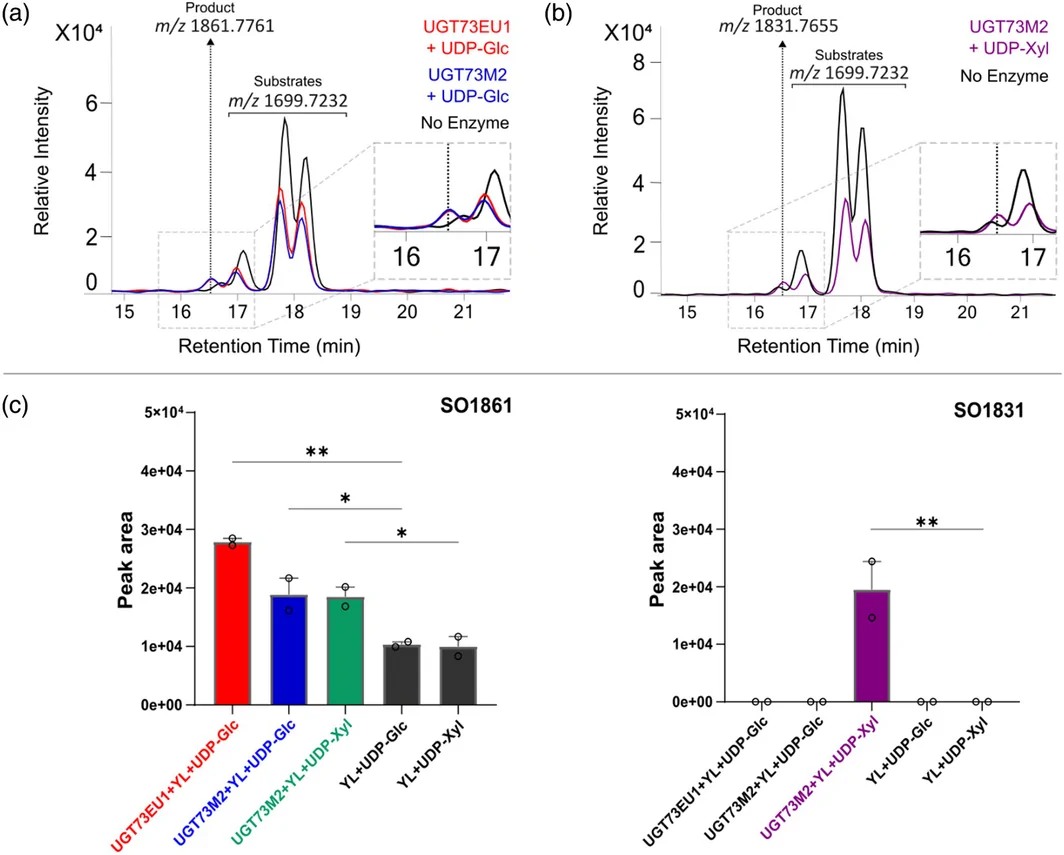
*In vitro* assays of UGT73EU1 and UGT73M2 with *Saponaria officinalis* young leaf extract. Overlayed extracted ion chromatograms (EICs) of substrate (SO1699, *m/z* 1699.7232) and products (SO1861, *m/z* 1861.7761 in (a); SO1831, *m/z* 1831.7655 in (b)) from LC–MS analysis of *in vitro* reactions, shown together to allow direct comparison of substrate consumption and product formation. Multiple peaks at the substrate *m/z* likely represent structural isomers naturally co‐occurring in the crude *S. officinalis* leaf extract. MS spectra of detected products are provided in Figure S10a–c. (c) Peak intensities obtained from LC–MS profiles for the products SO1861 (left side) and SO1831 (right side) after *in vitro* reactions using *S. officinalis* young leaves extracts (YL) incubated with recombinant UGT73EU1 and UGT73M2 in the presence of a UDP‐sugar donor. SO1861 was identified by comparison with authentic standard. Each bar represents the mean of two independent samples, and error bars indicate standard error of the mean; black circles display individual data points. *p < 0.05, **p < 0.01 (two‐tailed Student's *t*‐test).

To assess the biotechnological potential of UGT73EU1 and UGT73M2 to enhance SO1861 production using plant biomass as a source of metabolic precursors, we tested their activity on crude metabolite extracts from *S. officinalis*. Here, leaf extracts were selected as the substrate because they are reported to contain the relevant precursors in sufficiently high amounts, thus pointing to incomplete endogenous conversion and to the possibility of improving SO1861 yields *ex planta* (Jo et al., 2025). Thus, when methanolic extracts from *Saponaria* leaves were supplemented with UDP‐Glc, UGT73EU1 generated greater amounts of a compound compatible with SO1861 (hereafter *m/z* 1861) compared to UGT73M2 (Figure 6c; Figure S10a,b), matching the results obtained earlier in *in vitro* assays with purified precursors. Due to its low abundance, no MS/MS fragmentation was retrieved for this compound, making its assignment as SO1861 tentative. By contrast, UGT73M2 activity with crude extracts was mainly influenced by the type of sugar donor supplied. Although fragmentation data could similarly not be obtained for the *m/z* 1831 metabolite due to its low abundance, LC–MS analysis confirmed the presence of an SO1831‐compatible metabolite in leaf extracts incubated with UGT73M2 and supplemented with UDP‐Xyl (Figure S10c), in line with results from *in vitro* assays using purified substrates. Accordingly, supplementation of the leaf extract with UDP‐Glc led to a further accumulation of the *m/z* 1861 compound in the UGT73M2 reactions (Figure 6a; Figure S10b). An SO1861‐compatible *m/z* was also detected, albeit at lower abundance, in samples lacking UDP‐Glc supplementation (Figure 6c), likely reflecting the presence of SO1861 in the crude leaf extract. In conclusion, these experiments indicate that both UGT73EU1 and UGT73M2 can enhance levels of SO1861‐compatible compounds when applied directly to *S. officinalis* crude extracts.

### *UGT73EU1* overexpression in *G. elegans* hairy roots increases accumulation of an *m/z* 1861 compound

**Figure 7:**
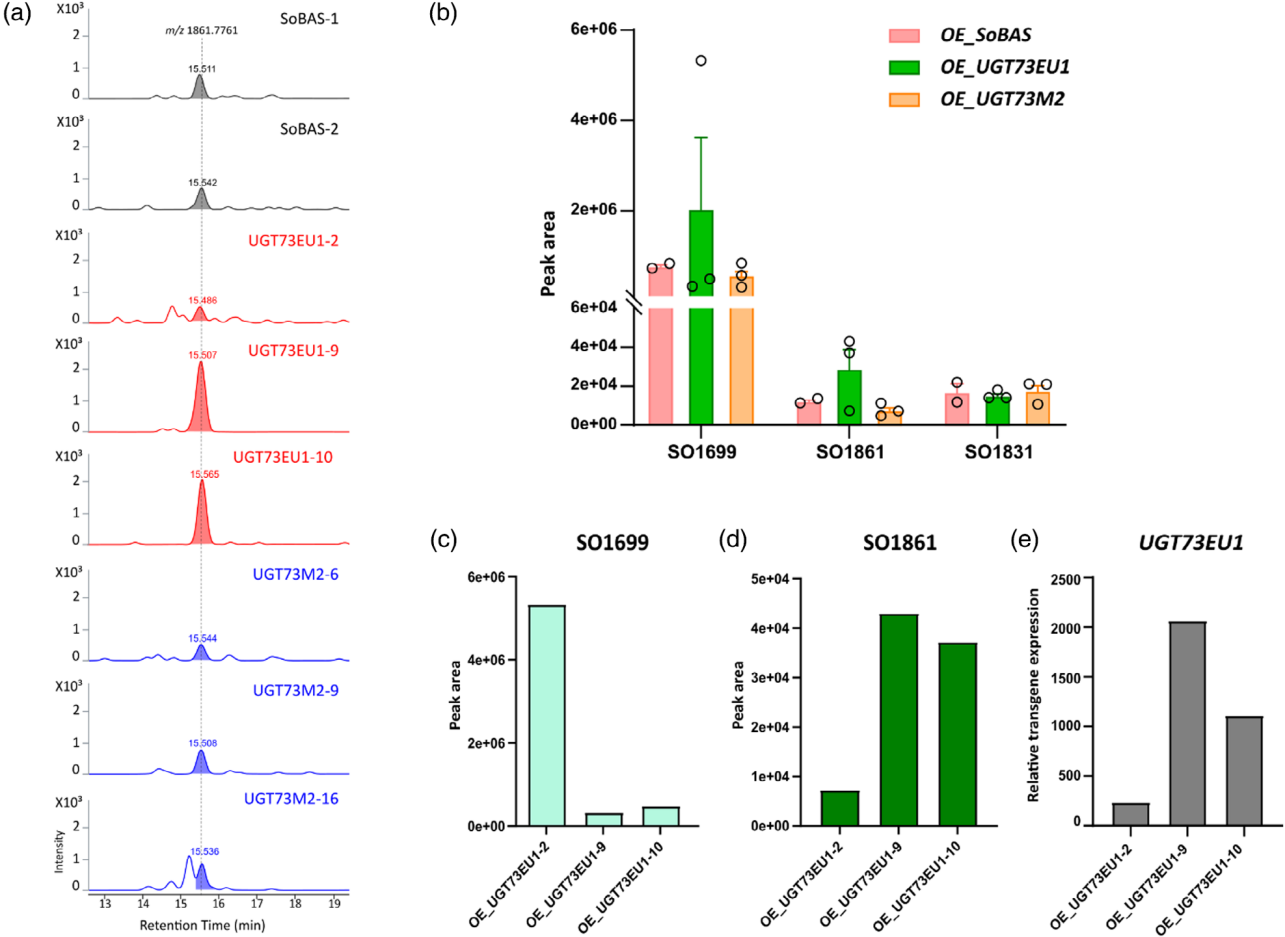
Expression of *UGT73EU1* in *Gypsophila elegans* hairy root cultures improves SO1861 accumulation. (a) Extracted ion chromatogram (EIC) of sapofectosid (SO1861, *m/z* 1861.7761) in individual transgenic *G. elegans* hairy root lines expressing *SoBAS* (control), *UGT73EU1*, or *UGT73M2*. The *in planta* SO1861 peak fell below the MS/MS selection threshold; its identity was assigned based on comparison to the SO1861 standard (Figure S10), in line with the MS/MS‐validated *in vitro* conversion of SO1699 into SO1861 by UGT73EU1 (Figure S5c). (b) Relative quantification of products SO1861 and SO1831, and their precursor SO1699 in transgenic *G. elegans* hairy root lines expressing *SoBAS*, *UGT73EU1*, or *UGT73M2*. (c–e) Peaks area of the precursor SO1699 (c) and the product SO1861 (d) and relative expression levels of *UGT73EU1* determined by RT–qPCR (e) in individual *UGT73EU1* overexpression lines. Glycyrrhizin was used as an internal standard to control for saponin extraction efficiency. Bars represent mean values of biological replicates (*n* = 2 for OE_SoBAS; *n* = 3 for OE_UGT73EU1 and OE_UGT73M2), and error bars indicate the standard error of the mean. In panels (c–e), individual *UGT73EU1*‐overexpression lines show a consistent relationship between higher *UGT73EU1* transcript levels, reduced accumulation of the precursor SO1699, and increased accumulation of the product SO1861.

To determine if our two lead UGTs could also impact the levels of SO1861 and related saponins *in planta*, we generated stably transformed *G. elegans* hairy root lines constitutively expressing *UGT73EU1* or *UGT73M2*. Hairy root lines expressing *SoBAS* were used as a control. While *SoBAS*‐expressing lines may present a metabolically primed pool of triterpene aglycones rather than a wild‐type baseline, we reasoned that any contribution of *SoBAS* overexpression to the late‐pathway flux is expected to be marginal compared to the direct action of late UGTs (Lacchini et al., 2025). Although we initially succeeded in generating *S. officinalis* hairy root lines expressing these genes, these lines exhibited weak transgene expression and slow growth, ultimately leading to unviable transgenic lines, which could not be cultivated. Hence, as in our previous work (Lacchini et al., 2025), we selected *G. elegans* hairy roots as our *in planta* platform given the demonstrated utility of these root cultures for plant sapofectosid production. We had previously demonstrated that *G. elegans* hairy root cultures possess the endogenous enzymatic machinery and precursor molecules normally used for gypsophilosid (i.e., GE1741) biosynthesis, that allow channeling of the metabolic flux toward SO1861 formation upon heterologous expression of key *Saponaria* enzymes‐related compounds, consistent with candidate precursors of gypsophilosid production (Lacchini et al., 2025; Sama et al., 2018). In fact, these reports suggested gypsophilosid and SO1861 to share common structural features and biosynthetic precursors. Here, to establish a consistent background normalization, we generated *G. elegans* hairy root lines constitutively expressing *eGFP* as a visual selection marker and as reference for relative expression of transgenes, together with either *UGT73EU1* or *UGT73M2*. Quantitative real‐time PCR results were thus used to confirm levels of transgenes expression (Figure S11c). LC–MS analysis of metabolic extracts from selected hairy root lines revealed that compared with *SoBAS* controls, lines overexpressing *UGT73EU1* accumulated higher levels of an *m/z* 1861 compound compatible with SO1861 (Figure 7b). Because the abundance of this peak in hairy root extracts was close to the signal‐to‐noise level of the instrument and below the standard MS/MS precursor selection threshold, a targeted MS/MS method was developed (see Methods section). This retrieved a fragmentation spectrum of the *in planta m/z* 1861 compound whose diagnostic ions (i.e., *m/z* 1729, corresponding to the loss of a terminal pentose; and *m/z* 955, corresponding to the C‐3 monodesmoside) matched those of the SO1861 standard (Figure 7a; Figure S12). This seems supported also by the MS/MS‐validated *in vitro* conversion of SO1699 into SO1861 by UGT73EU1 (Figure S5c). Full structural confirmation *in planta* would require NMR‐grade purification of the metabolite, which was hampered by its low abundance and the complexity of the saponin background. In particular, *OE_UGT73EU1_9* and *OE_UGT73EU1_10* displayed 3.5‐fold and threefold increase in total *m/z* 1861 content, respectively (Figure 7d), whereas no increase in total *m/z* 1861 content was observed in *OE_UGT73EU1_2* (Figure 7d), consistent with its lower transgene expression (Figure 7e). These increases correlated with a reduction in the presumed SO1861‐precursor, SO1699, which displayed an opposite pattern and thus reduced levels in *OE_UGT73EU1_9* and *OE_UGT73EU1_10* lines (Figure 7c). In line with *in vitro* (Figure 3) and kinetic studies (Figure S9), the enhanced levels of *m/z* 1861 in *OE_UGT73EU1* lines therefore likely reflect the enzyme's exclusive activity toward UDP‐Glc as donor and a narrower set of late‐pathway acceptors, concentrating its catalytic output on the formation of an SO1861‐compatible product and supporting a prominent role of UGT73EU1 *in planta*. By contrast, we did not observe an increased abundance of the *m/z* 1861 compound in the *UGT73M2*‐overexpressing lines (Figure 7b), despite *in vitro* assays confirming its ability to glucosylate SO1699, both with the purified acceptor (Figure 4a) and with crude leaf extracts (Figure 6a). Similarly, SO1831‐like *m/z* levels remained unchanged in *UGT73M2*‐overexpressing lines as compared to the *SoBAS* control lines (Figure 7b), despite its robust xylosylation activity *in vitro* (Figure 4b; Figures S6 and S7). The relatively high apparent Km of UGT73M2 for UDP‐Xyl, demonstrated by the kinetic assay (Figure S9d,f) may have limited the production of SO1831 in this heterologous context. In conclusion, *UGT73EU1*, appears as a useful genetic tool to drive the production of SO1861‐like saponins in *G. elegans* hairy root cultures, whereas *UGT73M2*, despite its strong *in vitro* activity on SO1831 production using purified precursors, appears less effective.

## DISCUSSION

SO1861 is a triterpenoid saponin with promising pharmaceutical applications as an EEE in targeted therapies. It is currently extracted mainly from the roots of *S. officinalis* plants (Masilamani et al., 2023; Schulze et al., 2024). Recently, the biosynthetic genes of two other structurally related saponins from *S. officinalis* were elucidated: SpB and its isomer SO1699, the latter being structurally coherent with a potential SO1861 precursor (Jo et al., 2025). However, the UGTs responsible for the final glycosylation step leading to SO1861 had not yet been identified. In this study, we identified UGT73EU1 as a terminal UGT in the SO1861 pathway and broadened the catalytic repertoire of UGT73M2, with both enzymes able to complete the last glycosylation step from its precursors *in vitro*.

Candidate UGTs involved in *S. officinalis* SO1861 biosynthesis were identified based on expression profile similarity with saponarioside biosynthetic genes, multiple amino acid sequence alignment, and functional characterization. A broad set of UGT candidates with similar expression profiles was screened through this combined approach, but only UGT73EU1 and UGT73M2 showed confirmed detectable catalytic activity toward SO1861 precursors, underscoring the specificity of these enzymes for this biosynthetic step. Protein sequence comparison revealed that UGT73EU1 and UGT73M2 cluster with functionally characterized UGTs known to mediate glycosylation in triterpenoid saponin biosynthesis. Within this cluster, UGT73M2 groups specifically with UGTs from *Q. saponaria* saponins, which glycosylate saponins sharing striking structural similarities with SO1861. *In vitro* enzymatic assays performed with recombinant proteins revealed that both *S. officinalis* UGTs, UGT73EU1 and UGT73M2, are active on the C‐28 position catalyzing the addition of a glucose moiety to yield SO1861. We previously characterized UGT73M2 by heterologous expression in *N. benthamiana* leaves as a xylosyltransferase responsible for the addition of terminal d‐xylose to the C‐28 branched sugar chain leading to SpB production (Jo et al., 2025). In this work, applying complementary assays, by *in vitro* enzymatic assays using either purified precursors (i.e., SO1699 and SO1730) or *S. officinalis* leaf extract as substrates, we showed that UGT73M2, in this context, can also produce SpA‐matching *m/z* compounds by adding d‐xylose on the C‐28 sugar chain of SO1699, another abundant saponin in *S. officinalis*. Notably, our results further support the role of UGT73M2 in utilizing also d‐glucose as sugar donor, facilitating the conversion of SO1699 into SO1861, expanding its functional versatility. The sugar donor flexibility of UGT73M2, in contrast with UGT73EU1, which primarily utilizes glucose, highlights differences but also a previously unrecognized functional overlap as both enzymes appear to glucosylate the same substrate, SO1699, to form SO1861. Together, these findings indicate that UGT73M2 may be a substantial contributor to the structural diversification of the complex saponin profile of *S. officinalis*. More broadly, they emphasize how the functional redundancy and substrate promiscuity of UGTs enable the production of structurally related molecules in plant tissues, thereby increasing their metabolic complexity (Lanier et al., 2023; Wu et al., 2022).

To further test potential biotechnological applications, in an alternative strategy, we showed the utility of recombinant UGT73EU1, and to a lesser extent UGT73M2, to convert SO1861 precursors present in crude metabolic extracts of soapwort leaves into products with features compatible with SO1861. Although these reactions yielded relatively low‐abundance products, the results exemplify both the nonoptimal flux through the pathway and the potential of incorporating an additional enzymatic step in an SO1861 production pipeline prior to purification. In our study, we produced recombinant UGTs in cell‐free lysates prepared from tobacco BY‐2 suspension cells using *in vitro* transcription and translation (Buntru et al., 2015, 2022), as these plant‐derived lysates are a performant platform for the expression of functional plant enzymes that are often challenging to obtain in bacterial hosts.

Given that hairy root cultures are considered as a powerful tool to produce high‐value secondary metabolites (Bagal et al., 2023; Li & Wang, 2021; Shi et al., 2021), we generated transformed hairy root cultures of *G. elegans* as the heterologous host for expression of our lead UGTs. *Gypsophila elegans* hairy roots are known to produce SO1861‐related compounds that could constitute a pool of precursors for SO1861 production by heterologous soapwort UGTs (Lacchini et al., 2025). Here, we also detected low levels of SO1861‐compatible *m/z* in *G. elegans* hairy roots overexpressing *Saponaria* β‐amyrin synthase, consistent with our previous findings (Lacchini et al., 2025) that upregulation of early‐pathway backbone genes can promote a ‘spill‐over’ of precursors into otherwise silent secondary, low‐efficient branches such as the SO1861 pathway. SO1861 itself is not a documented natural metabolite of wild‐type *G*. *elegans*. However, the close structural relationship between gypsophilosid and SO1861 and the QA‐based scaffold shared by saponins reported in *Gypsophila* species indicate that the precursor pool and upstream enzymatic machinery are likely compatible with a reconstituted SO1861 biosynthetic route. Thereby, engineering *G. elegans* hairy roots by *UGT73EU1* overexpression of *UGT73EU1* may have allowed a metabolic ‘pull’ that increased the accumulation of a SO1861‐compatible metabolite. While this *in planta* assignment remained tentative in the absence of MS/MS or NMR confirmation, it was consistent with the MS/MS‐validated activity of UGT73EU1 *in vitro*, where the enzyme displayed the desired exclusive activity toward SO1861 production from SO1699 substrate. These observations may thus point to a presumable role for UGT73EU1 in the late steps of the saponin biosynthetic pathway and indicate its potential as a tool for engineering the production of high‐value saponins in heterologous plant platforms.

Conversely, *UGT73M2*‐overexpressing hairy root lines did not increase the production of SO1861‐ or SO1831‐compatible compounds, despite the enzyme showing both glucosyltransferase and xylosyltransferase activity *in vitro*. This discrepancy likely arises from differences between *in vitro* enzyme assays, where purified substrates and enzymes are present at relatively high concentrations, and the complex *in planta* environment, where substrate availability is limited and shaped by metabolic fluxes, competition among structurally similar substrates, and overlapping enzyme activities. Our characterization therefore relied on the triangulation of three complementary readouts: UDP‐Glo™ screening to flag candidate activities, LC–MS of *in vitro* reactions to confirm product identities, and heterologous expression in hairy roots to evaluate physiological relevance *in planta*. The UGT73M2 glucosylation of SO1699 was supported by the first two layers of evidence but not the third, consistent with a promiscuous activity revealed under permissive *in vitro* conditions rather than a dominant role *in planta*. Furthermore, kinetics profiling indicates that the divergent activity *in planta* between the two enzymes is unlikely explained by acceptor affinity alone as both UGT73M2 and UGT73EU1 displayed low apparent Km values toward SO1699 that are presumably below physiological concentrations. Instead, differences between UGT73EU1 and UGT73M2 activity in hairy roots may be linked to the broader donor and acceptor plasticity of UGT73M2 displayed *in vitro*, confirmed by its markedly higher kcat with UDP‐Xyl, which may distribute its activity across multiple competing reactions *in vivo*, including its well established xylosyltransferase role upstream in the saponarioside pathway (Jo et al., 2025). Concomitantly, the relatively high apparent Km of UGT73M2 for UDP‐Xyl (155.7 μM) likely exceeds the cellular UDP‐Xyl concentration in plants, which in Arabidopsis has been estimated to reach only the low micromolar range (Ebert et al., 2015). This may further have limited the production of SO1831 in this heterologous context. UGT73EU1, by contrast, has shown restricted activity toward UDP‐Glc and a narrower set of late‐pathway acceptors *in vitro* so that its catalytic output can be concentrated on the formation of SO1861‐compatible products. This focused activity may be consistent with the observed accumulation in *UGT73EU1*‐overexpressing hairy root lines of a compound compatible with SO1861 presumably resulting from SO1699 glucosylation. The different kinetic properties of UGT73EU1 and UGT73M2 serve thus as a reminder that enzyme promiscuity and redundancy can result in distinct functional relevance depending on the application, the system, or the cellular context. These findings, while clarifying the division of labor between closely related UGTs, highlight a broader strategy for metabolic engineering of complex and intertwined pathways. Hence, by selecting heterologous host species that offer both efficient genetic transformability and an endogenous metabolic profile compatible with the target biosynthetic route, such as the natural production of precursors and structurally related intermediates, it becomes possible to reconstruct target pathways by heterologously expressing only one or a minimal set of key enzymes.

## METHODS

### RNA extraction and analysis

Total RNA was extracted from three different *S. officinalis* organs, each sampled from three clonal individuals, using the ReliaPrep RNA Miniprep System (Promega™, Madison, WI, USA). The extraction protocol and data analysis were performed as previously described (Lacchini et al., 2025).

### Phylogenetic analysis

Reference sequences of characterized plant triterpenoid/steroidal UGTs were retrieved from the UniProt database. Multiple protein sequence alignment was performed using the software MUSCLE, and phylogenetic analysis was carried out using IQ‐TREE (v2.4.0) (Minh et al., 2020) on usegalaxy.eu. ModelFinder was used to identify the best‐fit substitution model and subsequently applied in maximum likelihood (ML) tree reconstruction. Branch support was evaluated using the ultrafast bootstrap approximation (UFBoot) with 1000 replicates (Hoang et al., 2018).

### Enzyme expression in *E. coli*

To express and purify the recombinant protein for *in vitro* reaction and kinetic analysis, each gene was cloned into the pDEST17 Gateway vector encoding an N‐terminal 6× His tag (https://vectorvault.vib.be/), and these plasmids were transformed into *E. coli* BL21 cells. A bacterial pre‐culture was grown overnight in 5 ml of LB medium supplemented with carbenicillin (100 µg ml⁻¹) at 37°C at 200 rpm in an orbital shaker. This pre‐culture was then inoculated into 500 ml of LB medium at 37°C until the OD_600_ reached 0.6. Protein expression was induced by adding 100 μM isopropyl β‐d‐thiogalactoside (IPTG), followed by further incubation at 28°C at 200 rpm for 20 h. Cells were harvested by centrifugation at 6000× ***g*** for 10 min, then resuspended and lysed in Buffer A1 (50 mM Tris–HCl, 300 mM NaCl, 20 mM imidazole, and 10% (v/v) glycerol, pH 7.5) supplemented with EDTA‐free protease inhibitor tablets (Roche™, Basel, Switzerland). The lysate was sonicated in an ice bath and subsequently centrifuged at 20 000× ***g*** for 30 min at 4°C and the supernatant was placed with 500 μl of Ni‐NTA agarose beads (Takara™, Shiga, Japan), pre‐equilibrated in A1 buffer at 4°C for 1 h. The mixture was then passed through an A1‐primed PD‐10 column. The beads sedimented at the bottom of the column were washed twice with 10 ml of A1 buffer. Bound proteins were then eluted using 1 ml of A2 buffer (50 mM Tris–HCl, 200 mM NaCl, pH 7.5) with a stepwise imidazole gradient of 80 and 200 mM. The eluted proteins were concentrated using Amicon 30‐kDa size‐exclusion concentrators (Millipore™, Burlington, MA, USA). The purity of UGTs was confirmed by SDS‐PAGE, and the concentration of protein was determined using a NanoDrop 2000 spectrophotometer (Thermo Scientific, Waltham, MA, USA).

### Enzyme expression in BYL cell‐free lysate

The candidate UGTs from *S. officinalis* were cloned into the pLenEx vector (Buntru et al., 2022) and produced by *in vitro* transcription and translation using a cell‐free lysate (BYL) derived from *Nicotiana tabacum* cv. BY‐2 cells. Specifically, 3.9–4.9 ml BYL and 6.5–9.5 μg vector DNA were used for production of all the candidate UGTs. Cell‐free biosynthesis was performed in 96‐well plates (150 μl reaction per well) for 2 days.

For purification of the cell‐free produced UGTs, the BYL was centrifuged twice at 16 000× ***g*** for 5 min, transferring the supernatant to a fresh Eppendorf tube each time. The supernatant was then loaded onto a 1 ml Strep‐Tactin®XT Superflow® high‐capacity column (IBA Lifesciences, Göttingen, Germany) that had been equilibrated with 1× washing buffer (100 mM Tris pH 8.0, 150 mM NaCl, 1 mM EDTA). The column was washed with 6 ml 1× washing buffer, and elution was performed with 3 ml BXT buffer (100 mM Tris pH 8.0, 150 mM NaCl, 1 mM EDTA, 50 mM biotin) of which the first 0.5 ml was discarded. The purified UGTs were dialyzed using a slide‐A‐Lyzer G2 cassette (cutoff 10 kDa, size 3 ml; Thermo Fisher Scientific) (2× 2 L; overnight and 2 h at 4°C in cold room) or Vivaspin 6 centrifugal concentrators (cutoff 10 000 MWCO; Sartorius, Göttingen, Germany) against 100 mM Tris pH 8.0, 150 mM NaCl. When the centrifugal concentrators were used, the purified UGTs were also concentrated in the same step. The purified and dialyzed UGTs were analyzed by SDS–PAGE on precast NuPAGE 4–12% (w/v) polyacrylamide Bis‐Tris gels (Thermo Fisher Scientific) alongside PageRuler Prestained Protein Ladder markers (Thermo Fisher Scientific). The gels were stained with Coomassie Brilliant Blue R‐250. Determination of protein concentration was done photometrically at 280 nm and densitometrically using the Coomassie‐stained SDS–PAGE.

### *In vitro* enzymatic assay and kinetic analysis

To test the catalytic activity of candidate UGTs with different substrates, the UGT reaction assay was qualitatively screened using UDP‐Glo™ glycosyltransferase assay (Promega, Madison, WI, USA). Briefly, the assay relies on the fact that upon enzymatic addition of UDP‐sugar moiety by UGTs onto a compatible substrate, free UDP is released in the medium. Subsequently, the kit's reagents convert UDP into ATP, which is utilized by the enzyme Luciferase (provided with the kit) to transform d‐luciferin into oxyluciferin, generating light. The quantity of luminescence emitted during this process correlates directly with the amount of glycosylated product, and it can be quantified using a luminescence detector (Promega GlowMax®, Madison, WI, USA). The no‐(acceptor) substrate control was considered the blank reaction. All analytical standards as well as the saponin biosynthetic precursors of SO1861 used in the *in vitro* assays were purified to analytical grade from natural sources and provided by Extrasynthese (Genay, France).

To verify the function of candidate UGTs, the reaction mixture contained 20 mM Tris–HCl, 15 mM MgCl_2_ (pH = 7.5) buffer, 50 μg of purified recombinant protein, 100 μM SO1699 or SO1730 and 100 μM UDP‐Glc or UDP‐Xyl in a final volume of 100 μl. Assay conditions were optimized. Initial trials using 5 μg of protein with 100 μM UDP‐Glc and 200 μM SO1699 and incubated for 1.5 h at 30°C did not yield detectable product by LC–MS. Instead, product formation was resolved only after extending incubation to overnight. Subsequently, the reaction was stopped by adding methanol with 100 μM glycyrrhizin as an internal standard and centrifuged at 17 000× ***g*** for 30 min for protein precipitation. The supernatant was collected and sent for LC–MS analysis to the EdinOmics facility at the University of Edinburgh.

To determine the kinetic properties of recombinant UGT73EU1 and UGT73M2, the enzymatic assays were carried out in a total volume of 25 μl containing 20 mM Tris–HCl, 15 mM MgCl_2_ (pH 7.5) buffer, 600 ng purified protein, 100 μM of either UDP‐Glc or UDP‐Xyl with varying substrate concentrations. Protein concentrations for this assay were empirically determined to reduce background noise, thus maximizing sensitivity. The enzymatic reactions were incubated at 30°C for 1 h and then quenched with UDP‐Glo reagents, and luminescence was recorded 1 h later as specified by the manufacturer's instructions. Enzyme kinetics were determined using GraphPad Prism software.

### Subcellular localization and confocal microscopy analysis in *N. benthamiana* leaves

The coding sequences of candidate UGTs fused to a C‐terminal GFP encoding sequence were cloned into the Golden Gate vectors driven by the 35S promoter (https://vectorvault.vib.be/). Fusion constructs were transiently co‐expressed with an ER marker (Blue fluorescent protein; BFP‐HDEL) and cytosolic mCherry in *N. benthamiana* epidermal cells. Three days post‐infiltration, leaf discs were collected and analyzed for fluorescence using a Leica SP8X confocal microscope. BFP fluorescence was detected with 388 nm excitation and 440–450 nm emission wavelength. The GFP signal was detected with excitation at 488 nm and emission at 498–552 nm, while the RFP signal was detected with excitation at 561 nm and emission at 596–650 nm.

### Cultivation and transformation of *G. elegans* hairy roots

To investigate the function of UGTs *in planta* full length coding sequences with stop codons of UGT73EU1 and UGT73M2 were cloned into pK7WG2D (containing eGFP as a visible marker for transformation of the target gene in the hairy root) using Gateway technology (https://vectorvault.vib.be/). All constructs were transformed into *Agrobacterium rhizogenes* LBA9402. The hairy roots were generated from *G. elegans* composite plants, following the previous protocol for *M. truncatula* with minor modifications as previously reported (Pollier et al., 2011). The transformed *A. rhizogenes* were grown on solid YEB medium supplemented with spectinomycin (100 μg ml^−1^) and rifampicin (50 μg ml^−1^) at 28°C for 2 days. Around 10‐day‐old seedlings of *G. elegans* were cut about 2 mm from the root tip of the radicle and the wounded seedlings were dipped in transgenic *A. rhizogenes* cultures. The infected seedlings were cocultured on Murashige and Skoog solid medium containing vitamins (Duchefa, Haarlem, The Netherlands) for 10 days and transferred onto Murashige and Skoog solid medium containing 100 mg ml^−1^ cefotaxime for about 4 weeks at 25°C in the dark. Positive hairy roots were selected based on GFP fluorescence using a fluorescence microscope (Leica, Wetzlar, Germany) and further validated by RT‐qPCR. These positive hairy roots were cultured on an orbital shaker in Murashige and Skoog liquid medium supplemented with 1.5% (w/v) sucrose for 4 weeks in the dark at 25°C with shaking at 130 rpm and then hairy roots were collected for metabolite analysis.

### Quantitative real‐time PCR

Total RNA was isolated from ground transgenic *G. elegans* hairy roots with the ReliaPrep™ RNA Miniprep Systems (Promega™, Madison, WI, USA) following the manufacturer's instructions. Reverse transcription was performed using qScript cDNA SuperMix (QuantaBio™, Beverly, MA, USA). Reverse transcription‐quantitative polymerase chain reaction (RT‐qPCR) was performed with the Lightcycler 480 system (Roche, Basel, Switzerland) and analyzed with the Fast SYBR Green Master Mix (Thermo Fisher Scientific, Waltham, MA, USA). The PCR program included an initial denaturation at 95°C for 5 min, followed by 45 cycles consisting of denaturation at 95°C for 10 sec, annealing at 60°C for 15 sec, and extension at 72°C for 15 sec. Gene‐specific primers were designed with the Primer BLAST software (National Center for Biotechnology Information, NCBI). Oligonucleotides used in the RT‐qPCR analysis can be found in Table S2.

### Metabolite extraction

Frozen plant material (1 g fresh weight) was ground to a fine powder in liquid nitrogen and spiked with 1 ml of 1 mM glycyrrhizin as an internal standard. Saponins were extracted twice sequentially with 10 ml of 90% (v/v) methanol, each for 1 h on a rotary wheel. After each round, the samples were centrifuged at >13 500× *g* for 5 min and the two supernatants were pooled. The pooled extract was lyophilized overnight in a vacuum concentrator (SpeedVac). The dried residue was resuspended in 2 ml of milliQ water and subjected to liquid–liquid partitioning with hexane to remove nonpolar compounds: 2 ml of hexane was added, the mixture was vortexed until the residue was fully dissolved and centrifuged at 6700× ***g*** for 2 min, and the lower aqueous phase was collected. The retained hexane phase was re‐extracted twice more, each time with 2 ml of milliQ water, and the three aqueous fractions were pooled (approximately 6 ml total). The pooled aqueous extract was concentrated in a vacuum concentrator (SpeedVac) to a final volume of approximately 400 μl prior to LC–MS analysis.

### Liquid chromatography–mass spectrometry analysis

The saponin profiles were analyzed using liquid chromatography (LC) coupled to ion mobility (IM) quadrupole time‐of‐flight (qTOF) mass spectrometry (MS). The instrumentation consisted of an Agilent 1290 Infinity II series UHPLC system hyphenated with an Agilent 6560 IM‐qTOF with a Dual Agilent Jet Stream Electron Ionization source. LC separation was performed on a ZORBAX Extend‐C18 column (Agilent Technologies, Santa Clara, CA, USA). A 29‐min gradient was run using the organic buffer B (acetonitrile, 0.1% (v/v) formate) combined with the aqueous buffer A (water, 0.1% (v/v) formate). Data were acquired using MassHunter Data Acquisition 10.0 software on 5 μl of sample separated on the column with a flow rate of 300 μl min^−1^. The LC gradient consisted of 99% buffer A at min 0, 50% buffer A at min 20, 30% buffer A at min 25, 0% buffer A at min 27, and returned to 99% buffer A at min 28 where it was maintained for 1 min. Data were acquired in negative ionization mode between 50 and 3200 *m/z* range, with an MS acquisition rate of 1 spectra sec^−1^ and MS/MS acquisition rate of 3 spectra sec^−1^. A fixed collision energy of 40 V was used with a maximum of two precursors per cycle. The raw data files were visualized using the Agilent MassHunter Qualitative Analysis B.10.00 software and extracted ion chromatograms were obtained from MS level 1 scan with symmetric *m/z* ± 0.5.

For targeted detection of SO1861 in concentrated *G. elegans* hairy root extracts, a targeted MS/MS method was developed based on the LC–MS/MS method described above. The MS scan range was set to *m/z* 1800–1900, and the MS/MS scan range was set to *m/z* 500–1900. The collision energy was fixed at 20 V. The targeted method included monitoring of the precursor ion at *m/z* 1861.7761 using a medium isolation width (approximately 4 *m/z*).

## ACCESSION NUMBERS

All data are included in the article, Supporting Information, and/or public databanks. RNA‐Seq data have been deposited in the ArrayExpress database (accession E‐MTAB‐14773). The UGT73M2 sequences were already available on NCBI GenBank (Identity number OR426400), whereas the UGT73EU1 sequences have been deposited to GenBank, and given the Identity number PZ213937.

## AUTHOR CONTRIBUTIONS

TQ designed and performed experiments and analyzed data and wrote the manuscript; AG initiated, supervised the project, and wrote the manuscript; EL designed and performed experiments, analyzed data, supervised the project, and wrote the manuscript; HS designed and performed experiments, analyzed data, and contributed to writing the manuscript; TM designed and performed experiments and analyzed data; KR designed and performed experiments; RV performed experiments and purified analytical standards; SS designed experiments and analyzed data; all authors discussed the results and commented on the manuscript.

## CONFLICT OF INTEREST

The authors declare no competing interests.

## Supporting information


**Figure S1.** Comparison of two EEE‐type saponins: (a) sapofectosid from *S. officinalis* (i.e., SO1861, C_83_H_130_C_46_) and (b) gypsophilosid from *G. elegans* (i.e., GE1741, C_78_H_122_O_42_).
**Figure S2.** Proposed late steps in the sapofectosid biosynthetic pathway.
**Figure S3.** Purified recombinant *S. officinalis* UGTs for *in vitro* assays.
**Figure S4.** *In vitro* enzyme activity with UDP‐glucose.
**Figure S5.** MS and MS/MS characterization of substrates, products, and authentic standards from *in vitro* enzymatic assays with UDP‐glucose.
**Figure S6.** *In vitro* enzyme activity with UDP‐xylose.
**Figure S7.** *In vitro* enzyme activity with UDP‐xylose.
**Figure S8.** Confocal images of GFP‐translational fusions of previously reported UGTs involved in the biosynthesis pathway of SO1861 in Jo et al. (2024), transiently expressed in *N. benthamiana* leaves.
**Figure S9.** Kinetic analysis of recombinant UGT73EU1 and UGT73M2 toward their acceptor substrates and sugar donors.
**Figure S10.** MS spectra of SO1861 and SO1831 detected in *in vitro* assays with *S. officinalis* young leaf extracts.
**Figure S11.** qPCR analysis of transgenic *G. elegans* hairy root lines expressing *SoBAS*, *UGT73EU1*, and *UGT73M2*.
**Figure S12.** MS(/MS) spectra of SO1861 in *G. elegans* hairy root extracts.
**Table S1.** Overview of the outcome of the LC‐MS analysis of in vitro assays.
**Table S2.** Oligonucleotides used in this study.

## Data Availability

The data that support the findings of this study are openly available in ArrayExpress at https://www.ebi.ac.uk/biostudies/ArrayExpress/studies/E‐MTAB‐14773, reference number E‐MTAB‐14773.
