# Supplementary material for: Harnessing two UDP‐glycosyltransferases from Saponaria officinalis toward heterologous production of endosomal escape enhancer saponins in Gypsophila elegans hairy roots
Source: Plant J. 2026 Sep 22;127(6):e71109. doi: 10.1111/tpj.71109 (PMC13595893; doi:10.1111/tpj.71109)
Supplement: Supplementary file 1 [file TPJ-127-0-s001.docx]

SUPPLEMENTAL INFORMATION TO

Harnessing two UDP-glycosyltransferases from *Saponaria officinalis* towards heterologous production of endosomal escape enhancer saponins in *Gypsophila elegans* hairy roots

Tongtong Qu^1,2^, Helga Schinkel^3^, Tessa Moses^4^, Kaylan Reddy^1,2^, René de Vaumas^5^, Stefan Schillberg^3,6^, Alain Goossens^1,2,7,*^, and Elia Lacchini^1,2,8,*^

^1^ Department of Plant Biotechnology and Bioinformatics, Ghent University, 9052 Ghent, Belgium

^2^ VIB Center for Plant Systems Biology, 9052 Ghent, Belgium

^3^ Fraunhofer Institute for Molecular Biology and Applied Ecology IME, Forckenbeckstrasse 6, 52074 Aachen, Germany

^4^ EdinOmics, RRID:SCR_021838, University of Edinburgh, Max Born Crescent, Edinburgh, United Kingdom

^5^ Extrasynthese, Impasse Jacquard, 69730 Genay, France

^6^ Institute for Molecular Biotechnology, RWTH Aachen University, Worringerweg 1, 52074 Aachen, Germany

^7^ Department of Botany and Zoology, Stellenbosch University, Stellenbosch, South Africa

^8^ Department of Plant Molecular Biology, University of Lausanne, 1015 Lausanne, Switzerland

*Corresponding authors: [alain.goossens@psb.vib-ugent.be](mailto:alain.goossens@psb.vib-ugent.be); [elia.lacchini@unil.ch](mailto:elia.lacchini@unil.ch).

Figure S1: Comparison of two EEE-type saponins: (a) sapofectosid from *S. officinalis* (i.e., SO1861, C_83_H_130_C_46_) and (b) gypsophilosid from *G. elegans* (i.e., GE1741, C_78_H_122_O_42_) (Sama et al., 2018; Sama et al., 2017). Structural differences between SO1861 and GE1741 are highlighted in red.

Figure S2: Proposed late steps in the sapofectosid biosynthetic pathway.

Figure S3: Purified recombinant *S. officinalis* UGTs for *in vitro* assays. Five UGT proteins (UGT73M2, UGT73EU1, UGT75AM1, UGT74M2 and UGT80B35) were expressed using the tobacco BY-2 cell-free expression system, while the remaining three (UGT73A81, UGT73DN3 and UGT73A82) were heterologously expressed and purified from *E. coli*. Protein expression and purification were confirmed by SDS–PAGE.

Figure S4*: In vitro* enzyme activity with UDP-glucose. (a) Extracted ion chromatograms of substrate SO1699 and product SO1861 peaks in the presence of cofactor UDP-glucose and UGT73EU1 or UGT73M2. Standard of SO1861 was included. X-axis shows time in minutes and Y-axis corresponds to peak intensity of 1×10^6^. (b) Extracted ion chromatograms of MS/MS product ion 955.4535, expected to be present in both the substrate (peak at 16.1 min) and the product (peak at 15.1 min). The SO1861 authentic standard exhibited multiple peaks at the target *m/z*, indicating the presence of isomeric impurities or related sapogenin derivatives. In black is reported No UGT control (SO1699 substrate), in red UGT73EU1 reaction, in blue UGT73M2 reaction, in purple the SO1861 standard control.

Figure S5: MS and MS/MS characterization of substrates, products, and authentic standards from *in vitro* enzymatic assays with UDP-glucose. (a) MS and MS/MS fragmentation of authentic SO1699 standard at 40V collision energy. (b) MS and MS/MS fragmentation of authentic SO1861 standard at 40V collision energy. MS and MS/MS fragmentation of SO1861 produced by UGT73EU1 (c) and by UGT73M2 (d) at 40V. Fragmentation patterns of enzymatic products match those of the corresponding standards. In the mass spectra, red diamonds indicate the targeted precursor ions, while blue diamonds in the MS/MS spectra denote the m/z of the isolated parent ions selected for fragmentation.

Figure S6: *In vitro* enzyme activity with UDP-xylose. (a) Extracted ion chromatograms of substrate SO1699 and product xylosylated SO1700 peaks in the presence of cofactor UDP-xylose and UGT73M2. X-axis shows time in minutes and Y-axis corresponds to peak intensity of 6×10^5^ for product and 1.5×10^6^ for substrate. (b) Extracted ion chromatograms of MS/MS product ion 955.4535, which matches the quillaic acid C-3 trisaccharide monodesmoside expected to be present in both the substrate (peak at 16.1 min) and the product (peak at 15.1 min). In black is reported No UGT control (SO1699 substrate), in red UGT73M2 reaction.

Figure S7: *In vitro* enzyme activity with UDP-xylose. MS and MS/MS fragmentation of product SO1831 at 40V in reaction with SO1699 as substrate and UDP-Xyl in presence of UGT73M2. In the mass spectra, red diamonds indicate the targeted precursor ions, while blue diamonds in the MS/MS spectra denote the m/z of the isolated parent ions selected for fragmentation.

Figure S8: Confocal images of GFP-translational fusions of previously reported UGTs involved in the biosynthesis pathway of SO1861 in Jo *et al* (2024), transiently expressed in *N. benthamiana* leaves. Scale bars, 20 µm.

Figure S9: Kinetic analysis of recombinant UGT73EU1 and UGT73M2 towards their acceptor substrates and sugar donors. The enzymatic activity of UGT73EU1 was assessed with SO1699 (a) or SO1730 (b) as acceptor at varying concentrations, with UDP-Glc as the sugar donor. UGT73M2 activity was evaluated using SO1699 as the acceptor substrate in the presence of UDP-Glc, with enzyme kinetics determined by varying the concentrations of SO1699 (c) and UDP-Glc (d). In parallel, UGT73M2 activity was analyzed using UDP-Xyl as the sugar donor, with kinetic parameters determined by varying the concentrations of SO1699 (e) and UDP-Xyl (f). Data represent mean ± SEM from three technical replicates. Michaelis-Menten plots were fitted using nonlinear regression in GraphPad Prism. Apparent Km and kcat values were derived from best-fit curves.

Figure S10: MS spectra of SO1861 and SO1831 detected in *in vitro* assays with *S. officinalis* young leaf extracts. (a) SO1861 (m/z 1861.7761) in leaf extract incubated with UGT73EU1 and UDP-glucose. (b) SO1861 in leaf extract incubated with UGT73M2 and UDP-glucose. (c) SO1831 (m/z 1831.7655) in leaf extract incubated with UGT73M2 and UDP-xylose. In all panels, peak abundance fell below the MS/MS precursor selection threshold; compound assignments were therefore based on accurate mass and retention time, in line with the MS/MS-validated identifications obtained from *in vitro* assays with purified substrates (Figure S5 and S6).

Figure S11: qPCR analysis of transgenic *G. elegans* hairy root lines expressing *SoBAS*, *UGT73EU1* and *UGT73M2*. The relative expression was normalized using lines solely expressing GFP as a control. Each bar represents the mean of biological samples (two replicates for *OE_SOBAS*, and three replicates for *OE_UGT73EU1* and *OE_UGT73M2*), and error bars indicate standard error of the mean. All transformed root lines expressed *GFP* to similar levels, in accordance with its use as a visual transformation marker.

Figure S12: MS(/MS) spectra of SO1861 in *G. elegans* hairy root extracts. MS (a, c) and MS/MS (b, d) spectra of SO1861 in *G. elegans* hairy roots overexpressing *UGT73EU1* (*UGT73EU1*-OE) (c, d) and of the SO1861 standard (a, b), acquired at 20V collision energy using a targeted MS/MS method. In the MS/MS spectra of both the SO1861 standard (b) and the UGT73EU1-OE hairy-root sample (d), the two diagnostic fragment ions are observed: the quillaic acid C-3 trisaccharide monodesmoside (m/z 955; red arrow) and the loss of a terminal pentose (m/z 1729; green arrow). The inset in (b) maps these fragments onto the SO1861 structure, indicating the corresponding cleavage sites and resulting m/z. Blue diamonds indicate the *m/z* of isolated parent ions selected for MS/MS fragmentation.

| Enzyme | Cofactor | Substrate | Product  SO1831 | | |
| --- | --- | --- | --- | --- | --- |
|  |  |  | SO1861 | SO1831 | SO1891 |
| - | UDP-Glucose | SO1699 |  |  |  |
| UGT75AM1 |  |  |  |  |  |
| UGT80B35 |  |  |  |  |  |
| UGT73EU1 |  |  | x |  |  |
| UGT73M2 |  |  | x |  |  |
| UGT73A81 |  |  |  |  |  |
| UGT74M2 |  |  |  |  |  |
| UGT73DN3 |  |  |  |  |  |
| UGT73A82 |  |  |  |  |  |
| - | UDP-Xylose | SO1699 |  |  |  |
| UGT73M2 |  |  |  | x |  |
| - | UDP-Glucose | SO1730 |  |  |  |
| UGT75AM1 |  |  |  |  |  |
| UGT80B35 |  |  |  |  |  |
| UGT73EU1 |  |  |  |  | x |
| UGT73M2 |  |  |  |  |  |
| UGT73A81 |  |  |  |  |  |
| UGT74M2 |  |  |  |  |  |
| UGT73DN3 |  |  |  |  |  |
| UGT73A82 |  |  |  |  |  |

Table S1: Overview of the outcome of the LC-MS analysis of *in vitro* assays. Substrate and product specificity of the candidate UGTs is indicated. Boxes shaded in grey reflect the presence of the product in the specific combination of the acceptor substrate and UDP sugar donor.

| Target transcript | Oligo Sequence | Species | Notes |
| --- | --- | --- | --- |
| UGT73M2_F | GGGGACAAGTTTGTACAAAAAAGCAGGCTTAATGGAGGAATCAAAGGAGG | *Saponaria officinalis* | Gateway pDNR221 compatible ends |
| UGT73M2_R | GGGGACCACTTTGTACAAGAAAGCTGGGTCTCAAAATTTTTGTAGCACAGC | *Saponaria officinalis* | Gateway pDNR221 compatible ends |
| UGT73EU1_F | GGGGACAAGTTTGTACAAAAAAGCAGGCTTCATGGACTCGAATTCTAAC | *Saponaria officinalis* | Gateway pDNR221 compatible ends |
| UGT73EU1_R | GGGGACCACTTTGTACAAGAAAGCTGGGTCTTAGACTAGTTTTTGTGAC | *Saponaria officinalis* | Gateway pDNR221 compatible ends |
| UGT74M2_F | GGGGACAAGTTTGTACAAAAAAGCAGGCTTACGATCTCTTCACCAAAAATGGCTAA | *Saponaria officinalis* | Gateway pDNR221 compatible ends |
| UGT74M2_R | GGGGACCACTTTGTACAAGAAAGCTGGGTCTTAAGAAGATGAAAGCCACTCAATG | *Saponaria officinalis* | Gateway pDNR221 compatible ends |
| UGT75AM1_F | GGGGACAAGTTTGTACAAAAAAGCAGGCTCAATGGGAGGTCAACAAAAGC | *Saponaria officinalis* | Gateway pDNR221 compatible ends |
| UGT75AM1_R | GGGGACCACTTTGTACAAGAAAGCTGGGTATTAATGGATAGAATCACATAGGTTT | *Saponaria officinalis* | Gateway pDNR221 compatible ends |
| UGT73DN3_F | GGGGACAAGTTTGTACAAAAAAGCAGGCTTAATGGGTACTGAACCACAAAG | *Saponaria officinalis* | Gateway pDNR221 compatible ends |
| UGT73DN3_R | GGGGACCACTTTGTACAAGAAAGCTGGGTCTCAAGCATTATAATTGCTCA | *Saponaria officinalis* | Gateway pDNR221 compatible ends |
| UGT73A81_F | GGGGACAAGTTTGTACAAAAAAGCAGGCTTAATGGGTGCCGAATCTCGTCGT | *Saponaria officinalis* | Gateway pDNR221 compatible ends |
| UGT73A81_R | GGGGACCACTTTGTACAAGAAAGCTGGGTCTCATTTTTGAGAGTGATAG | *Saponaria officinalis* | Gateway pDNR221 compatible ends |
| UGT73A82_F | GGGGACCACTTTGTACAAGAAAGCTGGGTCTCATTTTTGAGAGTGATAG | *Saponaria officinalis* | Gateway pDNR221 compatible ends |
| UGT73A82_R | GGGGACAAGTTTGTACAAAAAAGCAGGCTTAATGGGTAGTAAACATCAACAC | *Saponaria officinalis* | Gateway pDNR221 compatible ends |
| UGT80B35_F | GGGGACAAGTTTGTACAAAAAAGCAGGCTTCATGGATAGTAGAGGAATTAA | *Saponaria officinalis* | Gateway pDNR221 compatible ends |
| UGT80B35_R | GGGGACCACTTTGTACAAGAAAGCTGGGTCCTATGAGGTTGAACAAACA | *Saponaria officinalis* | Gateway pDNR221 compatible ends |
| SoCSL1_F | AGAAGTGAAGCTTGGTCTCAGGCTCCATGTCACCCCACAACACCTG | *Saponaria officinalis* | pGGC Golden Gate compatible ends |
| SoCSL1_R | AGGGCGAGAATTCGGTCTCACTGAAGAGCGACCTTTTCTAGC | *Saponaria officinalis* | pGGC Golden Gate compatible ends |
| UGT73M2_F | AGAAGTGAAGCTTGGTCTCAGGCTCCATGGAGGAATCAAAGGAGG | *Saponaria officinalis* | pGGC Golden Gate compatible ends |
| UGT73M2_R | AGGGCGAGAATTCGGTCTCACTGAAAATTTTTGTAGCACAGC | *Saponaria officinalis* | pGGC Golden Gate compatible ends |
| UGT73EU1_F | AGAAGTGAAGCTTGGTCTCAGGCTCCATGGACTCGAATTCTAAC | *Saponaria officinalis* | pGGC Golden Gate compatible ends |
| UGT73EU1_R | AGGGCGAGAATTCGGTCTCACTGAGACTAGTTTTTGTGACAATTC | *Saponaria officinalis* | pGGC Golden Gate compatible ends |
| UGT73DL1_F | AGAAGTGAAGCTTGGTCTCAGGCTCCATGGGTTCAAATACAGAAGCAACT | *Saponaria officinalis* | pGGC Golden Gate compatible ends |
| UGT73DL1_R | AGGGCGAGAATTCGGTCTCACTGAAGCCTGCCTTAACGATC | *Saponaria officinalis* | pGGC Golden Gate compatible ends |
| UGT74CD1_F | AGAAGTGAAGCTTGGTCTCAGGCTCCATGTCGGATCAAAATGA | *Saponaria officinalis* | pGGC Golden Gate compatible ends |
| UGT74CD1_R | AGGGCGAGAATTCGGTCTCACTGAAAACCCACTCAATAATTTCGTC | *Saponaria officinalis* | pGGC Golden Gate compatible ends |
| UGT79L3_F | AGAAGTGAAGCTTGGTCTCAGGCTCCATGGGTACTAAAGAGTTAC | *Saponaria officinalis* | pGGC Golden Gate compatible ends |
| UGT79L3_R | AGGGCGAGAATTCGGTCTCACTGACTTCTCAACAAGATCTTG | *Saponaria officinalis* | pGGC Golden Gate compatible ends |
| GeUBQ10 F | TCGTTAAGACCTTGACTGGGC | *Gypsophila elegans* | qPCR |
| GeUBQ10 R | AATCAGCAAGGGTACGACCG | *Gypsophila elegans* | qPCR |
| SoBAS_F | TTGTGGCGATCTTCTATGGC | *Saponaria officinalis* | qPCR |
| SoBAS_R | AGGGCCGTGAGTAAGTTGA | *Saponaria officinalis* | qPCR |
| UGT73M2_F | CCGCTCTTTCTAGGCTCTGT | *Saponaria officinalis* | qPCR |
| UGT73M2_R | GTTGCATAACGACACTGGGC | *Saponaria officinalis* | qPCR |
| UGT73EU1_F | TCCCGAGTAAGGGACTCGAA | *Saponaria officinalis* | qPCR |
| UGT73EU1_R | GCACCCCATTGCTTAACCCT | *Saponaria officinalis* | qPCR |
| GFP_F | ATGGCCGACAAGCAGAAGAA | *Saponaria officinalis* | qPCR |
| GFP_R | CTCAGGTAGTGGTTGTCGGG | *Saponaria officinalis* | qPCR |
| UGT80B35_F | TTACATTCTACAACTACCATGGATAGTAGAGGAATTAATAATG |  | pLenEx cloning |
| UGT80B35_R | CAAACCAGAAGAGCTTGGTACCTTATTATTTTTCGAATTGCGGATGAGACCATGCGGCCGCTGAGGTTGAACAAACACAGC |  | pLenEx cloning |
| UGT74M2_F | TTACATTCTACAACTACCATGGCTAATGAAAACAATAC |  | pLenEx cloning |
| UGT74M2_R | CAAACCAGAAGAGCTTGGTACCTTATTATTTTTCGAATTGCGGATGAGACCATGCGGCCGCAGAAGATGAAAGCCACTCAATG |  | pLenEx cloning |
| UGT73EU1_F | TTACATTCTACAACTACCATGGACTCGAATTCTAAC |  | pLenEx cloning |
| UGT73EU1_R | CAAACCAGAAGAGCTTGGTACCTTATTATTTTTCGAATTGCGGATGAGACCATGCGGCCGCGACTAGTTTTTGTGAC |  | pLenEx cloning |
| UGT73M2_F | TTACATTCTACAACTACCATGGAGGAATCAAAGGAGGAAGTAC |  | pLenEx cloning |
| UGT73M2_R | CAAACCAGAAGAGCTTGGTACCTTATTATTTTTCGAATTGCGGATGAGACCATGCGGCCGCAAATTTTTGTAGCACAGCTTTGG |  | pLenEx cloning |
| UGT75AM1_F | TTACATTCTACAACTACCATGGGAGGTCAACAAAAGC |  | pLenEx cloning |
| UGT75AM1_R | CAAACCAGAAGAGCTTGGTACCTTATTATTTTTCGAATTGCGGATGAGACCATGCGGCCGCATGGATAGAATCACATAGG |  | pLenEx cloning |

Table S2: Oligonucleotides used in this study.

References

Jo, S., El-Demerdash, A., Owen, C., Srivastava, V., Wu, D., Kikuchi, S., Reed, J., Hodgson, H., Harkess, A., Shu, S., Plott, C., Jenkins, J., Williams, M., Boston, L.B., Lacchini, E., Qu, T., Goossens, A., Grimwood, J., Schmutz, J., Leebens-Mack, J. and Osbourn, A. (2025) Unlocking saponin biosynthesis in soapwort. *Nat Chem Biol* 21, 215-226.

Sama, S., Jerz, G., Schmieder, P., Joseph, J.F., Melzig, M.F. and Weng, A. (2018) Plant derived triterpenes from Gypsophila elegans M.Bieb. enable non-toxic delivery of gene loaded nanoplexes. *J Biotechnol* 284, 131-139.

Sama, S., Jerz, G., Schmieder, P., Woith, E., Melzig, M.F. and Weng, A. (2017) Sapofectosid - Ensuring non-toxic and effective DNA and RNA delivery. *Int J Pharm* 534, 195-205.
